# Investigating differential practice effects on cued task-order coordination and coordination adjustment in dual tasks

**DOI:** 10.3389/fpsyg.2026.1880062

**Published:** 2026-08-13

**Authors:** Nils Assink, Tilo Strobach

**Affiliations:** 1 Department of Psychology, MSH Medical School Hamburg, Hamburg, Germany; 2 ICAN Institute of Cognitive and Affective Neuroscience, MSH Medical School Hamburg, Hamburg, Germany

**Keywords:** cognitive control, coordination adjustment, dual tasks, practice, task-order coordination

## Abstract

**Introduction:**

Performing two overlapping tasks in dual-task situations requires task-order coordination, particularly when task order varies across trials. This coordination is associated with performance impairments on trials with different-order compared to same-order task sequences relative to the preceding trial (i.e., *order switch costs*). In addition, recent evidence suggests that task-order coordination can be adjusted based on contextual demands, as reflected in reduced order-switch costs following preceding different-order compared to same-order trials (i.e., *switch adjustment effects*). Practice effects provide an opportunity to disentangle these processes. However, previous research has largely relied on dual-task paradigms in which the task order is determined solely by the sequence of stimulus presentation, thereby limiting the role of top-down control via task-order cues.

**Methods:**

The present study investigates task-order coordination and coordination adjustment in a dual-task paradigm in which the order of a visual digit task and an auditory tone task is indicated by explicit cues. Across four sessions, dual-task and single-task practice effects were compared.

**Results:**

The results show reduced order-switch costs in Session 4 compared to Session 1, whereas no substantial changes were observed in the switch adjustment effects.

**Discussion:**

These findings are interpreted as practice-related improvements in task-order coordination, whereas evidence for practice effects on coordination adjustment remains limited.

## 1 Introduction

When two tasks are temporally overlapping under dual-task conditions, performance decrements usually occur in one or both of these tasks when compared with their separate task performance under single-task conditions (i.e., *dual-task costs*). One of the most widely used experimental paradigms to study such performance costs is the dual-task paradigm of the “Psychological Refractory Period” (PRP) type. In PRP dual tasks, a first and a second task are presented with varying intervals (i.e., stimulus-onset asynchronies; SOAs), and participants are instructed to make choice reaction time (RT) responses to both stimuli with a priority on the first task. RTs and/or error rates in the second task typically show an increase as SOAs decrease from long (simulating single-task conditions) to short, indicating the PRP effect (Koch et al., 2018; Pashler, 1994; Pashler and Johnston, 1989; Schubert, 2008; Strobach et al., 2015b).

**Figure 1:**
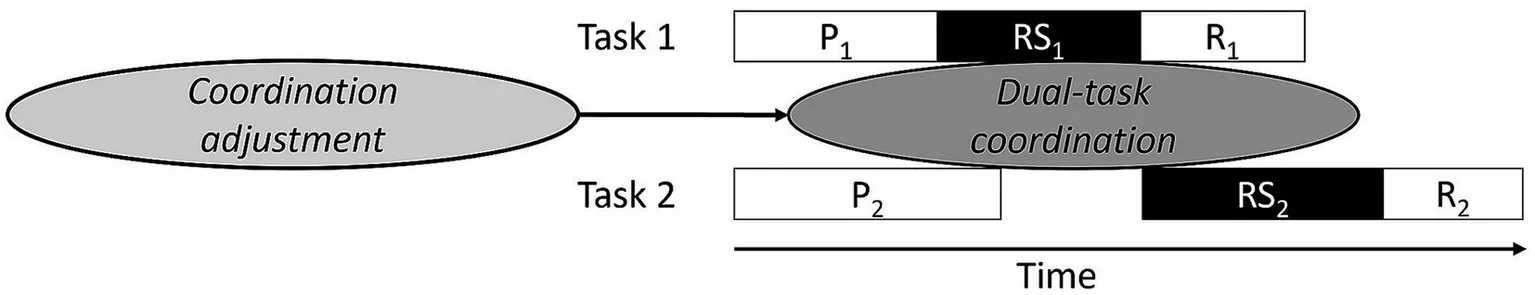
The framework of dual-task coordination and coordination adjustment illustrates the temporal relationship between the processing stages of Tasks 1 and 2 in a dual-task paradigm with a stimulus-onset asynchrony (SOA) of 0 ms. As shown, there is a bottleneck limitation in the processing of the tasks that prevents simultaneous execution of the response-selection stage (indicated in black). Processes of dual-task coordination are associated with the control of the scheduling of these bottleneck limitations, and dual-task coordination is conceptualized as distinct from the component tasks that constitute the dual task. Additional control allows for adjusting this coordination (i.e., dual-task coordination adjustment). Dual-task costs in terms of longer processing times compared to single tasks appear in Task 2 when task processing is interrupted between P_2_ and RS_2_ processing stages. Note that the illustrated locations of dual-task coordination and its adjustment are not explicitly related to the temporal relations between these levels. In the illustrated Tasks 1 and 2, P_1_ and P_2_ indicate the perception stages, RS_1_ and RS_2_ indicate the central response-selection stages (including capacity limitation), and R_1_ and R_2_ indicate the response-execution stages.

The PRP effect has been explained by capacity limitation models, which assume that two tasks share limited processing resources, preventing either task in dual-task situations from being performed as quickly as when executed alone (Hazeltine et al., 2006; Meyer and Kieras, 1997; Strobach, 2024a). One account, the capacity sharing model (Tombu and Jolicœur, 2003; Wu and Wang, 2024), proposes that the central processing stages of two tasks can proceed in parallel by sharing these limited resources. However, because the available capacity must be shared between tasks, processing efficiency is reduced, leading to dual-task costs. The specific strategy of a full distribution of processing resources to one component task and no such resources shared with the other task is consistent with the assumptions of the central bottleneck model (Pashler, 1994). According to this model, a full distribution of processing resources to one task is explained with a structural and unavoidable processing bottleneck limitation. This bottleneck limitation prevents the two tasks from being performed in parallel. As illustrated in Figure 1, while the perception and response-execution stages of the tasks can basically be processed in parallel, the PRP effect arises from a structural and unavoidable processing bottleneck limitation at the tasks’ response-selection stages. These stages cannot be processed simultaneously and must occur sequentially (McCann and Johnston, 1992; Pashler, 1994). Consequently, when the response selection of the first task is processed, the response selection of the second task has to wait and is postponed.

In order to regulate the scheduling of task processing at the bottleneck, cognitive control in the form of dual-task coordination is required (Logan and Gordon, 2001; Meyer and Kieras, 1997; Salvucci and Taatgen, 2008). This dual-task coordination is especially important when task order varies unpredictably, as random stimulus presentation also changes the processing order of response-selection stages at the bottleneck (Kübler et al., 2018; Stelzel et al., 2008; Strobach et al., 2015c; Strobach et al., 2021b; Szameitat et al., 2002, 2006). Moreover, recent studies have demonstrated that the required task-order coordination is flexibly adjusted through cognitive control in response to contextual demands, such that prior conflict experience enhances task-order coordination (for a review, see Strobach, 2024a). In addition to direct contextual influences, task-order coordination is also shaped by experience gained through practice. Differential practice-related effects on task-order coordination and its adjustment have been found, suggesting a practice-related dissociation between these two processes (Strobach, 2024b), a finding that will be further discussed in Section 1.2. However, these findings remain relatively underspecified, as they primarily focus on PRP dual-task paradigms in which task order is exclusively determined by the sequence of stimulus presentation. As a result, practice effects have mainly been investigated in situations where bottom-up information from stimulus presentation order is processed. In contrast, considerably less is known about practice effects when participants can use task-order cues to prepare and coordinate task order in advance. Therefore, the present study aims to examine practice effects on task-order coordination and coordination adjustment in dual tasks in which task order is signaled by task-order cues. Examining practice effects in a cue-based dual-task paradigm will help determine whether the previously observed dissociation between task-order coordination and task-order coordination adjustment (Strobach, 2024b) generalizes to situations in which participants can prepare task order in advance. This, in turn, will provide further insides into the cognitive mechanisms underlying these aspects of dual-task coordination.

### 1.1 Task-order coordination and task-order coordination adjustment in dual tasks

Several studies have used PRP paradigms with varying task orders, in which the task order is solely determined by the sequence of stimulus presentation. In doing so, they reported increased RTs and error rates in trials involving a switched task order relative to the previous trial (i.e., different-order trials) in comparison to trials in which the task order repeats from the previous trial (i.e., same-order trials). These performance costs on different-order compared to same-order trials, referred to as *order-switch costs*, suggest the involvement of active task-order coordination (Kübler et al., 2018; Strobach et al., 2021b; Strobach, 2024a; Szameitat et al., 2006). Order-switch costs are attributed to the need to establish a new task-order set when the task sequence changes. The implementation of a new task-order set, potentially accompanied by the inhibition of a previous active set, requires additional cognitive processing and therefore results in performance costs (Hirsch and Koch, 2024; Kübler et al., 2022a).

Moreover, these order-switch costs are also influenced by contextual demands, such as the recent processing history. According to Strobach (2024a), the order-switch costs in the current trial (Trial N) are adjusted by the order condition of the previous trial (Trial N-1). In particular, a number of studies (Strobach et al., 2021a, 2023; Strobach and Wendt, 2022) demonstrated that order-switch costs in Trial N are reduced when Trial N-1 constitutes a different-order rather than a same-order trial. A different-order Trial N-1 occurs when the task order in Trial N-1 differs from that in Trial N-2 (see Table 1, task-order sequences (3) and (4)), whereas a same-order Trial N-1 occurs when the task order remains unchanged from that in Trial N-2 (task-order sequences (1) and (2)). This sequential adjustment of order-switch costs is referred to as the *switch adjustment effect*. The switch adjustment effect was observed across both tasks that constitute the dual tasks, affecting RTs, error rates, and/or response reversal rates (i.e., the response order differs from the order of the stimulus presentation). This was evident across numerous conditions within PRP paradigms in which task order was determined solely by the sequence of stimulus presentation. These conditions included dual-task combinations with two-choice tasks and three-choice tasks, dual tasks with relatively short and long SOAs, dual tasks with relatively short and long intervals between trials, and dual tasks with differences in dominance between the two individual tasks as well as dual tasks without such differences (for an overview see Strobach, 2024a). In sum, this set of studies provided substantial evidence for processes of dual-task coordination (e.g., task-order coordination) and coordination adjustment, as conceptualized in the framework shown in Figure 1 (Strobach, 2024a). According to this framework, the *separability hypothesis* posits that dual-task coordination and coordination adjustment are distinct sets of processes, potentially indicated by differences in their attributes. For instance, while dual-task coordination is influenced by SOA (Strobach et al., 2021a), working memory load (Kübler et al., 2022b; Strobach et al., 2021a), intertrial interval (Strobach and Wendt, 2022), and task-order preferences (Huestegge et al., 2021; Strobach et al., 2023), dual-task coordination adjustment remains largely unaffected by these experimental factors.

**Table 1:** Overview of the different task-order sequences in a dual-task paradigm with tasks A and B in Trials N-2, N-1 and N, with the example of the task order AB in current Trial N.

Condition	Trial N-2	Trial N-1	Trial N	Task-order sequence
(1)	AB	AB	AB	Same-order ➔ Same-order
(2)	BA	BA	AB	Same-order ➔ Different-order
(3)	BA	AB	AB	Different-order ➔ Same-order
(4)	AB	BA	AB	Different-order ➔ Different-order

Reduced order-switch costs (i.e., the performance difference between a same-order and a different-order Trial N) between conditions 4 versus 3 in relation to the difference between conditions 2 versus 1 indicate the switch adjustment effect.

Next to the behavioral evidence of task-order coordination and coordination adjustment, Steinhauser et al. (2024) provided neurobehavioral evidence for these processes. The authors reanalyzed the data of two previously published experiments with PRP dual-task paradigms combining a visual task (e.g., digit identification) and an auditory task (e.g., tone identification). They did so with regard to whether order-switch costs and the corresponding electroencephalography (EEG) component (i.e., the order-switch positivity) in the current Trial N would be modulated by order switches versus repetitions in the previous Trial N-1. Compared with the aforementioned studies, another extension of the study of Steinhauser et al. (2024) was the use of a dual-task situation in which the order is not exclusively signaled by the order of the stimulus presentation. In addition to the presentation order, this order was indicated by a cue at the beginning of each trial, informing participants about the upcoming task sequence (with a cue-stimulus interval of 600 ms in Experiment 1 and 1,000 ms in Experiment 2). This order cue was particularly relevant because order processing based on the stimulus order presentation is extremely challenging in this particular dual task since this situation applied a relatively short SOA of 200 ms. As indicated by Töllner et al. (2012), such a short SOA level without order cues results in a relatively high number of trials in which participants respond in the wrong task order. Moreover, it is not only the bottom-up information of the stimulus presentation order that is processed exclusively, but there is also explicit order information from a cue preceding the stimulus presentation. The latter type of information leads to active, top-down task-order control processes that are often assumed to occupy a separate processing stage before the processing of the individual component tasks (De Jong, 1995; Kübler et al., 2018, 2019, 2022a, 2022b; Kürten et al., 2024; Luria and Meiran, 2003; Sigman and Dehaene, 2006; Strobach et al., 2021b). In the first experiment, Steinhauser et al. (2024) found a switch adjustment effect, particularly in RTs. In contrast, this effect was not evident in the behavioral data of Experiment 2, which was attributed to the longer cue-stimulus interval. Importantly, however, both experiments revealed a switch adjustment effect in the EEG data. That is, the order-switch positivity in Trial N was significantly reduced when Trial N-1 was a different-order trial compared to a same-order trial. Taken together, these findings provide initial behavioral and, in particular, neural evidence for task-order coordination and its adjustment in dual-task situations in which order cues are applied.

### 1.2 Practice effects on task-order coordination and task-order coordination adjustment in dual tasks

Numerous studies have shown that dual-task costs can be reduced with practice (Göthe et al., 2016; Hazeltine et al., 2002; Kamienkowski et al., 2011; Ruthruff et al., 2001). While some aspects of this reduction have been attributed to improvements within the individual component tasks themselves (i.e., single-task practice effects; Ahissar et al., 2001; Maquestiaux et al., 2008; Ruthruff et al., 2006; Sangals et al., 2007), evidence also suggests improvements in task-independent dual-task coordination (Liepelt et al., 2011; Schubert et al., 2017, 2024; Strobach et al., 2015a). This improvement of dual-task coordination can be achieved during dual-task practice with tasks practiced more or less simultaneously compared to the effects of single-task practice with tasks practiced separately under single-task conditions. Improved dual-task coordination is associated with dual-task coordination skills which reflect practice-related enhancements in coordinating two simultaneously presented and temporally overlapping tasks (Kramer et al., 1995; Maquestiaux et al., 2004). Because these skills are independent of the specific tasks practiced, they allow for a transfer from practiced dual tasks to unpracticed dual-task situations (Strobach, 2020).

There are two recent studies that empirically investigated improved dual-task coordination for the case of task-order coordination. Darnstaedt et al. (2024) conducted a 5-session practice regime with different dual-task practice conditions. Participants were assigned to practice of two visual-manual tasks either under a varying task-order condition or a fixed task-order condition. Before and after practice, both practice groups performed pre- and post-tests, respectively, including the practiced dual-task conditions and unpracticed dual-task transfer conditions, in which new tasks and task stimuli were presented. The results showed that varying task-order dual-task practice particularly improved task-order coordination for the practice and transfer conditions in comparison to fixed task-order dual-task practice. However, this study is limited in that it focused exclusively on dual-task practice and did not include a comparison with single-task practice effects, which would have been necessary to clearly identify the improvement of dual-task coordination skills.

Further, it is open whether dual-task practice also affects task-order coordination adjustment or not, which would provide conclusions regarding the separability hypothesis. To investigate this question, Strobach (2024b) conducted a visual–auditory PRP dual-task paradigm and compared performance after four sessions of dual-task practice with varying task orders to performance after single-task practice. The results demonstrated that (1) dual-task practice improved task-order coordination and (2) these improvements were greater than those observed after single-task practice. However, unlike task-order coordination itself, task-order coordination adjustment did not show clear improvements following dual-task practice. Thus, task-order coordination and coordination adjustment processes possess different characteristics regarding their practice-related sensitivity. This pattern suggests a practice-related dissociation between dual-task coordination and dual-task coordination adjustment, which is consistent with the separability hypothesis proposed in the framework shown in Figure 1.

### 1.3 The present study: testing practice effects on cued task-order coordination and coordination adjustment in dual tasks

Previous literature on dual-task coordination is merely at an infant stage in specifying the cognitive mechanisms underlying coordination adjustment (Strobach, 2024a). Despite aforementioned preliminary evidence, the cognitive research literature does not provide further empirical tests for the separability hypothesis in dual-task practice scenarios that require task-order coordination. Therefore, the main aim of the present study is to test the generalizability of the separability of task-order coordination and coordination adjustment as well as to further develop our understanding of these mechanisms in the context of dual-task practice.

Practice-related investigations of task-order coordination and coordination adjustment have predominantly applied dual-task scenarios in which task order is exclusively determined by the sequence of stimulus presentation. Consequently, only bottom-up information derived from stimulus order is processed, and any practice-related changes primarily affect the coordination of these processing streams. In contrast, the interaction between practice effects and active, top-down control of task order based on task-order cues (De Jong, 1995; Kübler et al., 2018, 2019, 2022a, 2022b; Kürten et al., 2024; Luria and Meiran, 2003; Sigman and Dehaene, 2006; Strobach et al., 2021b) has largely been neglected. Therefore, the present study applies the dual-task setting of Steinhauser et al. (2021, 2024; Experiment 1), in which the order of a visual digit task and an auditory tone task was indicated by an order cue (square versus diamond). By comparing dual-task and single-task practice effects across four sessions in a between-subject design, the present study investigates practice effects on task-order coordination and task-order coordination adjustment in order-cued dual tasks. Practice constitutes a particularly useful tool for testing the separability of these two processes, as differential practice-related effects would indicate distinct underlying mechanisms. Consistent with Strobach (2024b), evidence of improved task-order coordination in order-cued dual tasks following dual-task practice, contrasted with no improvement in task-order coordination adjustment, would support the generalizability of a practice-related dissociation and the separability hypothesis of dual-task coordination and coordination adjustment.

## 2 Materials and methods

### 2.1 Participants

Participants were students at the Medical School Hamburg and other universities in the Hamburg area. Recruitment was conducted using online databases and personal contacts. All participants were native German speakers and right-handed, as investigated with the Edinburgh Handedness Inventory (Oldfield, 1971); in this test, right-handedness is represented by a laterality quotient, ranging from 1 (very low right-handedness) to 100 (strong right-handedness). Participants reported normal or corrected-to-normal hearing and vision, provided informed consent prior to the start of the study, and received course credit for their participation. All procedures performed in this experiment were in accordance with the 1964 Helsinki declaration and its later amendments. The experiments were approved by the local ethics committee of the MSH Medical School Hamburg (reference number MSH-2023/282).

The recruitment period was between September 18, 2023, and September 30, 2024. Sample size computations were performed with G*Power software (Faul et al., 2007). The calculation for the within-subject comparison was based on the assumption of a possible interaction between CURRENT TRIAL, PREVIOUS TRIAL, and SESSION with an expected effect size of ηp2 = 0.24 [equivalent to *f* = 0.56, derived from Strobach (2024b)], which would indicate a dual-task practice effect on task-order coordination adjustment. The power analysis was conducted *a priori* for a repeated-measures ANOVA with the following parameters: *α* err prob.: 0.05; Power (1 − *β* err prob): 0.85; number of groups: 1; number of measurements: 2; corr. Among repeated measures: 0.00 (a conservative estimate, since higher correlations increase statistical power and reduce the required sample size) and nonsphericity correction *ε*: 1. This resulted in a required sample size of *N* = 17. The calculation for the between-subject comparison was based on the assumption of a possible interaction between CURRENT TRIAL and GROUP with an expected effect size of ηp2 = 0.15 [equivalent to *f* = 0.42, derived from Strobach (2024b)], which would indicate different practice effects on task-order coordination following dual-task compared to single-task practice.^1^ The power analysis was conducted a priori for a repeated-measures ANOVA with the following parameters: α err prob.: 0.05; Power (1 − *β* err prob): 0.85; number of groups: 2; number of measurements: 2; corr. Among repeated measures: 0.00 and nonsphericity correction ε: 1. This resulted in a required sample size of *N* = 14 per group. To address possible exclusions arising from low accuracy or comparable issues, we increased the sample size for the dual-task practice group to 27 (20 females, mean age: 22.11 years, age range: 19–34 years; mean handedness laterality quotient: 99.2, handedness laterality quotient range: 78–100) and for the single-task practice group to 24 (17 females, mean age: 22.00 years, age range: 18–27 years; mean handedness laterality quotient: 98.1, handedness laterality quotient range: 75–100).

### 2.2 Apparatus

Visual stimuli were presented on a 22-inch color monitor (refresh rate: 60 Hz, viewing distance: approximately 60 cm). Auditory stimuli were delivered via headphones connected to IBM-compatible personal computers. Experimental control and stimulus presentation were managed using the software package Presentation (Version 25). Manual responses were given on QWERTZ keyboards.

### 2.3 Stimuli

**Figure 2:**
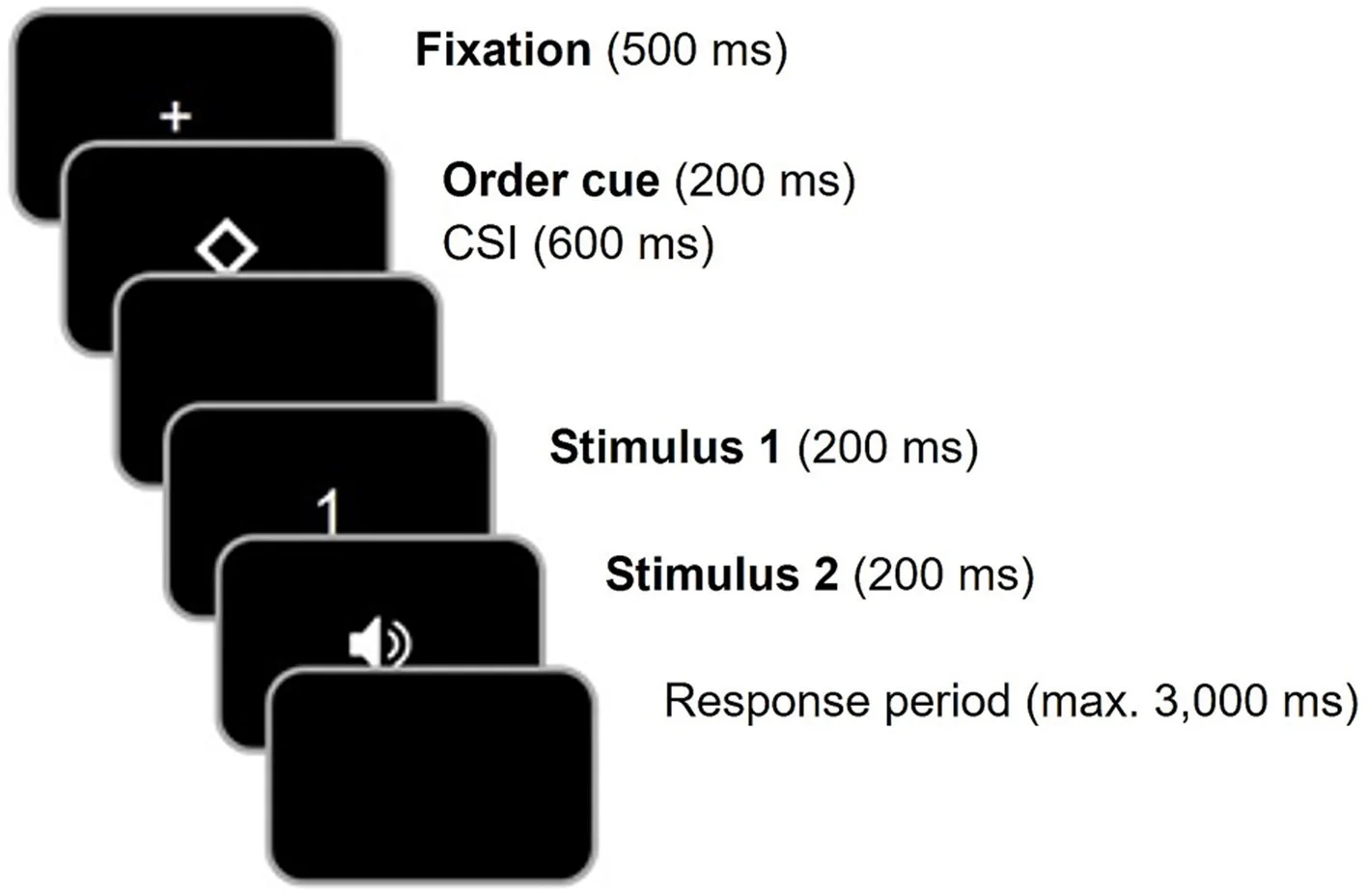
Illustration of a dual-task trial with a visual–auditory task order. Following a 500 ms fixation, an order cue was presented for 200 ms. After a 600 ms cue-stimulus interval (CSI), the stimuli are presented for 200 ms with a stimulus-onset asynchrony (SOA) of 200 ms. A response period of up to 3,000 ms followed, with the next trial starting 500 ms after the response to the second task. A dual-task trial with an auditory–visual task order was identical to this procedure, except that the stimulus presentation order was switched. Further, the timing of single-task trials was identical to the one in dual-task trials, except that no second stimulus was presented and the response period started immediately after the first stimulus.

Participants performed a dual-task situation consisting of one auditory and one visual discrimination task with random task order that was indicated by a visual cue (see Figure 2). The auditory task included the presentation of sine-wave tones with pitches of either 200, 650, or 1,100 Hz. Responses were given based on the tone pitch with the left-hand fingers – ring (Y key), middle (X key), and index (C key) fingers, respectively. The visual task included the presentation of the digits “1,” “5,” and “9,” and responses were given based on the identity with the right-hand fingers – index (, key), middle (. key), and ring (− key) fingers, respectively. The two isoluminant visual cues signaling the upcoming stimulus modality were either a square with a white outline (indicating that the next stimulus will be auditory) or a corresponding diamond (indicating that the next stimulus will be visual). The same auditory and visual stimuli were also used in the corresponding single-task conditions.

### 2.4 Procedure and design

Before Session 1, all participants completed an introduction phase consisting of four single-task blocks of 18 trials each to familiarize participants with the stimulus–response mappings, and six dual-task blocks of 18 trials each in the following order:

- Two single-task blocks with the visual task.
- Two single-task blocks with the auditory task.
- Two dual-task blocks with a visual–auditory task order.
- Two dual-task blocks with an auditory–visual task order.
- Two dual-task blocks with varying task orders.

Following the introduction phase, participants were randomly assigned to one of two groups: a dual-task practice or a single-task practice group.

Participants in the dual-task practice group completed ten dual-task blocks of 72 trials with varying task orders in each of Sessions 1 to 4 (720 dual-task trials per session). Sessions 2 to 4 followed the same procedure as Session 1 but did not include the introduction phase. At the beginning of each block, participants were instructed to respond to both tasks in the order indicated by the cue as quickly and accurately as possible. Each session lasted approximately 60 min.

Participants in the single-task practice group completed one dual-task block of 72 trials in Session 1. Performance in this block was compared with the corresponding block in the dual-task practice group to assess baseline dual-task performance before practice. Participants then practiced the component tasks separately. In Session 1, they completed 18 single-task blocks of 72 trials, whereas in Sessions 2 and 3 they completed 20 single-task blocks of 72 trials per session. Auditory and visual single-task blocks were administered in alternating order, beginning with the auditory task. This procedure ensured an equivalent number of stimulus exposures across groups prior to Session 4. In the final Session 4, participants of the single-task practice group completed the same dual-task protocol as the dual-task practice group, namely ten dual-task blocks of 72 trials with varying task orders (720 dual-task trials). Sessions 1 to 3 lasted approximately 90 min, whereas Session 4 matched the duration of the dual-task practice group.

Participants in both groups were allowed to take breaks between blocks. The introduction phase and Session 1 were completed on the same day, whereas Session 1 (inducing the introduction phase) to 4 took place on separate days within 1 week. Table 2 provides an overview of the block order across sessions and across groups.

**Table 2:** Overview of the block order in Session 1 to 4 in the dual-task (DT) practice group and in the single-task (ST) practice group.

Block number	Session 1		Session 2		Session 3		Session 4	
	DT practice group	ST practice group	DT practice group	ST practice group	DT practice group	ST practice group	DT practice group	ST practice group
1								
2								
3								
4								
5								
6								
7								
8								
9								
10								
11								
12								
13								
14								
15								
16								
17								
18								
19								
20								

Dual-task blocks with varying task orders are indicated by darker grey, whereas single-task blocks are indicated by brighter grey. Note that the first DT block in Session 1 in both groups were compared to assess baseline dual-task performance (see Section 3.2).

Each single-task trial in the single-task blocks began with the presentation of a fixation cross for 500 ms, followed by a cue for 200 ms and a cue-stimulus interval of 600 ms. A stimulus was then presented for 200 ms, followed by a black screen during the response period. The next trial began 500 ms after the response or a maximum response period of 3,000 ms. The timing of dual-task trials was identical to that of single-task trials, except that 200 ms after the onset of the first tasks stimulus, the stimulus for the second task was presented for 200 ms (SOA = 200 ms), before the response period began. In dual-task blocks with a visual–auditory order, the visual stimulus always preceded the auditory stimulus, whereas in blocks with an auditory–visual order, the auditory stimulus always preceded the visual stimulus. In dual-task blocks with varying task orders, presentation order was randomized and counterbalanced such that an equal number of trials began with the visual stimulus and the auditory stimulus. Errors (incorrect responses or omissions) triggered error feedback (German word: “Fehler”).

### 2.5 Data analyses

The first two trials of each dual-task block were discarded from the analyses because those trials do not have two previous trials to analyze the impact of the preceding task orders. For the within-subject comparison of Session 1 and Session 4 in the dual-task practice group (see Section 3.1), only participants with complete data and average error rates below 30% in each of Session 1 and Session 4 were included in the analysis (see Strobach et al., 2021a; Strobach and Wendt, 2022). These criterions ensured sufficient valid data for statistical analyses across both sessions. Three participants completed Session 1 but not Session 4, and seven participants were excluded due to exceeding the error-rate threshold in one or both sessions. This resulted in a final sample of 17 participants for this analysis (12 females, mean age: 21.76 years, age range: 19–28 years; mean handedness laterality quotient: 100.0). In contrast to the within-subject comparison of Session 1 and Session 4, the between-subject comparison of Session 4 performance focused exclusively on performance in Session 4 (see Section 3.2). Therefore, the inclusion criterion was applied only to Session 4, so that only participants with complete data and an average error rate below 30% in Session 4 were included (see Strobach et al., 2021a; Strobach and Wendt, 2022). Moreover, Session 1 involved different practice conditions across groups, making a Session 1 based exclusion criterion less appropriate for this comparison. This resulted in a final sample of 22 participants in the dual-task practice group (15 females, mean age: 21.86 years, age range: 19–28 years; mean handedness laterality quotient: 100.0) and 20 participants in the single-task practice group (15 females, mean age: 22.17 years, age range: 18–27 years; mean handedness laterality quotient: 97.8; handedness laterality quotient range: 75–100). The datasets from Session 2 and 3 are complete and can be accessed on Open Science Framework (OSF, https://osf.io/xfz5p/overview?view_only=6318f3d4c7634c7986d9b4919c4a97de), alongside the datasets from the other sessions.

RTs (after the exclusion of current trials including errors on the choice decisions within the component tasks and response reversals), error rates, and response reversal rates were aggregated in four trial conditions: a same-order Trial N following a same-order Trial N-1 (see task-order sequence (1) in Table 1, AB ➔ AB ➔ AB), a different-order Trial N following a same-order Trial N-1 (see task-order sequence (2) in Table 1, BA ➔ BA ➔ AB), a same-order Trial N following a different-order Trial N-1 (see task-order sequence (3) in Table 1, BA ➔ AB ➔ AB), as well as a different-order Trial N following a different-order Trial N-1 (see task-order sequence (4) in Table 1, AB ➔ BA ➔ AB). Although response reversal rates are part of the overall error rates, their separate analyses are additionally informative as they indicate failures to perform the instructed task order, reflecting limitations in sequential control and task scheduling in dual-task performance, despite the potential selection of correct responses for each component task. For the comparison between Session 1 and Session 4 in the dual-task practice group, these four trial conditions were entered in repeated-measures ANOVAs including the within-subject factors CURRENT TRIAL (same-order versus different-order trial in Trial N), PREVIOUS TRIAL (same-order versus different-order trial in Trial N-1), and SESSION (1 versus 4). Evidence for a practice effect on task-order coordination would be indicated by an interaction between CURRENT TRIAL and SESSION. Evidence for a practice effect on the adjustment of task-order coordination would be indicated by an interaction between CURRENT TRIAL, PREVIOUS TRIAL, and SESSION. For the comparison between Session 4 of the dual-task and single-task practice groups, the same four trial conditions were entered in mixed-measures ANOVAs including the within-subject factors CURRENT TRIAL (same-order versus different-order trial in Trial N) and PREVIOUS TRIAL (same-order versus different-order trial in Trial N-1), and the between-subject factor GROUP (dual-task versus single-task practice group). Evidence for an impact of the different learning conditions on task-order coordination would be manifested in an interaction between CURRENT TRIAL and GROUP. Evidence for an impact of the different learning conditions on the adjustment of task-order coordination would be manifested in an interaction between CURRENT TRIAL, PREVIOUS TRIAL, and GROUP. A first set of *post hoc t*-tests included comparisons of same-order and different-order trials at Trial N to assess order-switch costs in these trials as a function of the order condition in Trial N-1. In a second set of analyses, the effect of Trial N-1 was examined separately for same-order and different-order trials at Trial N. Comparing performances following same-order versus different-order Trial N-1 enables the investigation of adjustment patterns. Analyses were conducted for Task 1 (firstly presented task, irrespective of either auditory or visual task) and Task 2 (secondly presented task, irrespective of either auditory or visual task): RT1 (RT of Task 1), RT2 (RT of Task 2), Error1 (errors in Task 1) and Error2 (errors in Task 2). Response reversals could not be analyzed separately for the two tasks. Additionally, Bayes factors (BF_01_) were computed by comparing the BF_01_ value of the model including the factor or factor combination with that of the corresponding model without the factor or factor combination. While a resulting BF_01_ value smaller than 1 provides evidence in favor of the factor or factor combination, a value larger than 1 provides evidence for a model excluding the respective factor or factor combination (Van Den Bergh et al., 2020). A fixed seed was used to ensure reproducibility (seed = 20,457).

### 2.6 Transparency and openness

Data were generally analyzed using RStudio, version 2025.09.1 (Posit, 2025). Bayesian factors were computed using JASP (JASP Team, 2025). Supplementary material, including all data, the analysis code, and used packages is available at Open Science Framework (OSF, https://osf.io/xfz5p/overview?view_only=6318f3d4c7634c7986d9b4919c4a97de). Additional research materials can be provided upon request. This study was not preregistered.

## 3 Results

Along with the detailed results report in this section, there is an overview of the main findings in Table 3.

**Table 3:** Overview of available evidence on dual-task practice effects in task-order coordination and task-order coordination adjustment for the within-subject (Session 1 vs. Session 4) and between-subject (DT practice vs. ST practice group) comparisons in reaction times (RT1 and RT2), error rates (Error1 and Error2), and response reversal rates.

Comparison	Evidence on dual-task practice effects in cued…	
	…task-order coordination	…task-order coordination adjustment
Within-subject comparison (Session 1 vs. Session 4)	CURRENT TRIAL*SESSION	CURRENT TRIAL*PREVIOUS TRIAL*SESSION
RT1	**✓** (BF_01_ = 0.027)	–* (BF_01_ = 0.166)
RT2	**✓** (BF_01_ = 0.044)	–* (BF_01_ = 0.104)
Error1	**✓** (BF_01_ = 0.737)	– (BF_01_ = 1.391)
Error2	**✓** (BF_01_ = 0.299)	– (BF_01_ = 1.414)
Response reversals	**✓** (BF_01_ = 0.085)	– (BF_01_ = 1.733)
Between-subject comparison (DT practice vs. ST practice group)	CURRENT TRIAL*GROUP	CURRENT TRIAL*PREVIOUS TRIAL*GROUP
RT1	**✓** (BF_01_ = 0.249)	– (BF_01_ = 1.410)
RT2	**✓** (BF_01_ = 0.581)	–* (BF_01_ = 0.810)
Error1	– (BF_01_ = 1.270)	– (BF_01_ = 2.030)
Error2	– (BF_01_ = 1.520)	– (BF_01_ = 1.593)
Response reversals	– (BF_01_ = 3.251)	– (BF_01_ = 1.069)

Symbols indicating the presence (✓) or absence (–) of effects are based on frequentist analyses using the conventional 5% significance threshold (*p* < 0.05). Asterisks (*) indicate results for which the Bayesian analyses reveal reduced evidential strength. DT and ST refers to dual-task and single-task, respectively.

### 3.1 Within-subject comparison of Session 1 and Session 4 in the dual-task practice group

#### 3.1.1 Dual-task practice effects as reflected in RTs

**Figure 3:**
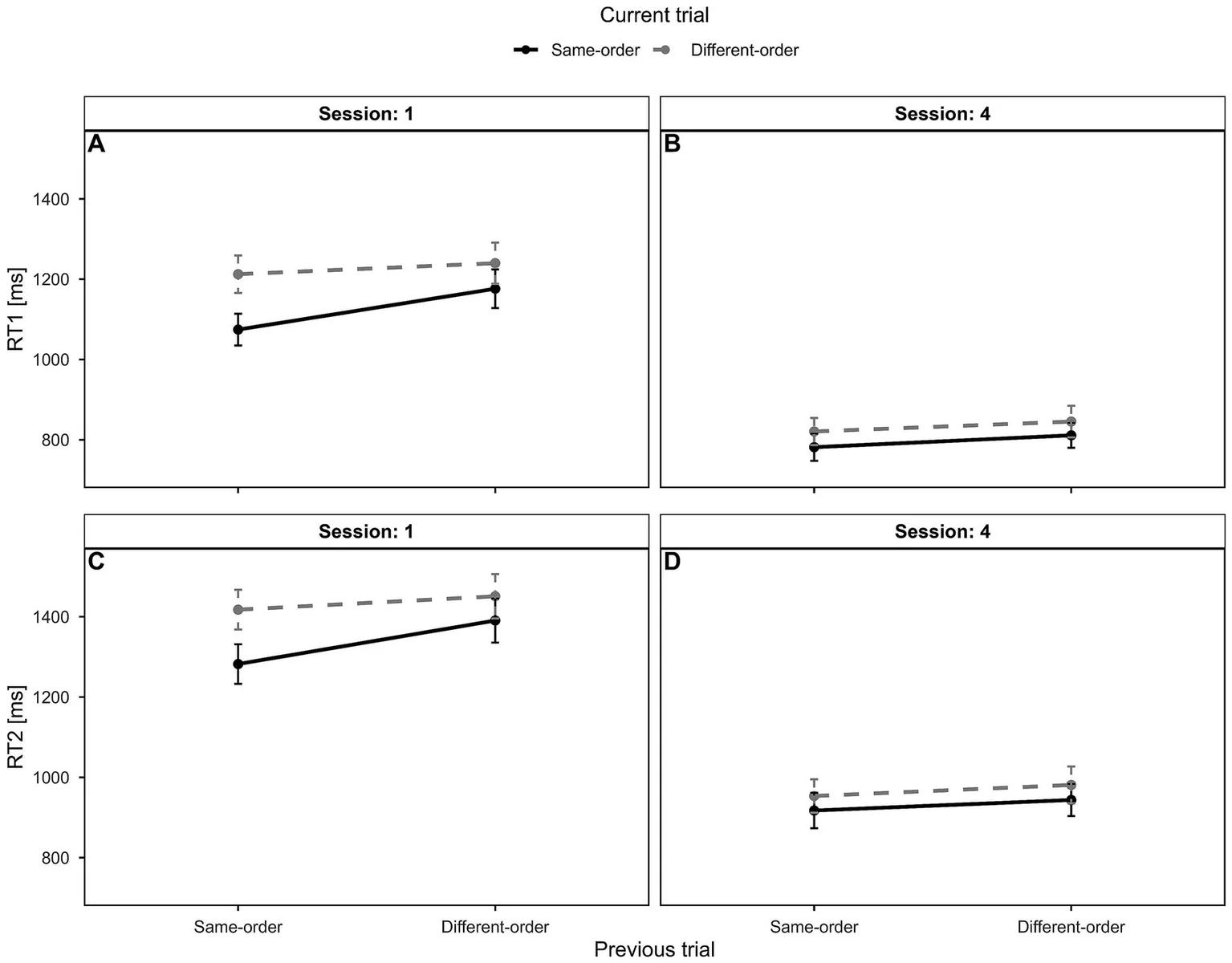
Reaction times in the first (RT1) and second task (RT2) in ms for Session 1 **(A,C)** and Session 4 **(B,D)** in the dual-task practice group. RTs are shown for same-order and different-order in the current trial and for same-order and different-order in the previous trial. Error bars represent standard errors of the mean.

The analysis of RT1 revealed a significant main effect of CURRENT TRIAL, *F*(1, 16) = 28.06, *p* < 0.001, *ŋp2* = 0.637, BF_01_ = 0.003, indicating order-switch costs (Kübler et al., 2018; Strobach, 2024a; Strobach et al., 2021b; Szameitat et al., 2006). As illustrated in Figures 3A,B, RTs were shorter in same-order trials (*M* = 961 ms, SD = 138 ms) compared to different-order trials (*M* = 1,030 ms, SD = 158 ms). A significant main effect of PREVIOUS TRIAL also emerged, *F*(1, 16) = 33.68, *p* < 0.001, *ŋp2* = 0.678, BF_01_ = 0.006, indicating generally shorter RTs in the current trials when the previous ones were same-order trials (*M* = 972 ms, SD = 140 ms) relative to different-order trials (*M* = 1,018 ms, SD = 153 ms). Additionally, a significant main effect of SESSION appeared, *F*(1, 16) = 99.61, *p* < 0.001, *ŋp2* = 0.862, BF_01_ < 0.001, reflecting general practice effects. RTs were overall shorter in Session 4 (*M* = 815 ms, SD = 139 ms) compared to Session 1 (*M* = 1,176 ms, SD = 185 ms). Furthermore, CURRENT TRIAL was modulated by SESSION, *F*(1, 16) = 39.78, *p* < 0.001, *ŋp2* = 0.713, BF_01_ = 0.027. Order-switch costs decreased from Session 1 (*M* = 101 ms; *t*(16) = −6.17, *p* < 0.001, *d* = −1.50) to Session 4 (*M* = 37 ms; *t*(16) = −3.33, *p* = 0.004, *d* = −0.81), indicating improved task-order coordination during dual-task practice (Strobach, 2024b). Moreover, the effect of PREVIOUS TRIAL was modulated by SESSION as well, *F*(1, 16) = 7.90, *p* = 0.013, *ŋp2* = 0.330, BF_01_ = 0.063, revealing a reduction in slowing down after previous different-order trials compared to same-order trials between Session 1 (*M* = 65 ms; *t*(16) = −5.01, *p* < 0.001, *d* = −1.22) and Session 4 (*M* = 27 ms; *t*(16) = −3.95, *p* = 0.001, *d* = −0.96). Importantly, RT1 showed a significant interaction between CURRENT TRIAL and PREVIOUS TRIAL, *F*(1, 16) = 8.83, *p* = 0.009, *ŋp2* = 0.355, BF_01_ = 0.504. Order-switch costs in the current trials were significantly smaller following different-order trials (*M* = 49 ms; *t*(33) = −4.53, *p* < 0.001, *d* = −0.78) compared to following same-order trials (*M* = 89 ms; *t*(33) = −5.28, *p* < 0.001, *d* = −0.91). Further, RTs in current same-order trials were significantly smaller when the previous trial were also same-order trials compared to different-order trials (*M* = 66 ms; *t*(33) = −5.79, *p* < 0.001, *d* = −0.99). Current different-order trials were also significantly smaller following same-order trials compared to different-order trials (*M* = 26 ms; *t*(33) = −2.17, *p* = 0.037, *d* = −0.37). However, the modulation by SESSION of this interaction did not reach statistical significance, *F*(1, 16) = 3.41, *p* = 0.083, BF_01_ = 0.166, although Bayesian analysis indicated only weak support for the null hypothesis.

The analysis of RT2 revealed similar results. The main effect of CURRENT TRIAL was significant, *F*(1, 16) = 27.52, *p* < 0.001, *ŋp2* = 0.632, BF_01_ = 0.003, suggesting the presence of order-switch costs. As depicted in Figures 3C,D, participants responded more quickly in same-order trials (*M* = 1,133 ms, SD = 176 ms) compared to different-order trials (*M* = 1,201 ms, SD = 180 ms). The effect of PREVIOUS TRIAL also emerged significance, *F*(1, 16) = 33.15, *p* < 0.001, *ŋp2* = 0.674, BF_01_ = 0.005, revealing generally shorter RTs in the current trials when the previous ones were same-order trials (*M* = 1,143 ms, SD = 173 ms) relative to different-order trials (*M* = 1,192 ms, SD = 181 ms). SESSION also exerted influence, *F*(1, 16) = 135.26, *p* < 0.001, *ŋp2* = 0.894, BF_01_ < 0.001, consistent with general practice effects. Responses were overall faster in Session 4 (*M* = 949 ms, SD = 174 ms) compared to Session 1 (*M* = 1,385 ms, SD = 209 ms). Moreover, CURRENT TRIAL was modulated by SESSION, *F*(1, 16) = 42.59, *p* < 0.001, *ŋp2* = 0.727, BF_01_ = 0.044. Consistent with the assumption of improved task-order coordination during practice, order-switch costs decreased from Session 1 (*M* = 98 ms; *t*(16) = −6.03, *p* < 0.001, *d* = −1.46) to Session 4 (*M* = 37 ms; *t*(16) = −3.51, *p* = 0.003, *d* = −0.85). Furthermore, the effect of PREVIOUS TRIAL was likewise modulated by SESSION, *F*(1, 16) = 9.44, *p* = 0.007, *ŋp2* = 0.371, BF_01_ = 0.412, showing that the slowing after previous different-order trials compared to same-order trials was reduced from Session 1 (*M* = 71 ms; *t*(16) = −4.99, *p* < 0.001, *d* = −1.21) to Session 4 (*M* = 27 ms; *t*(16) = −4.01, *p* = 0.001, *d* = −0.97). Finally, RT2 exhibited a significant interaction between CURRENT TRIAL and PREVIOUS TRIAL, *F*(1, 16) = 5.98, *p* = 0.026, *ŋp2* = 0.272, BF_01_ = 0.744. Order-switch costs were smaller following different-order trials (*M* = 49 ms; *t*(33) = −4.79, *p* < 0.001, *d* = −0.82) compared to following same-order trials (*M* = 86 ms; *t*(33) = −5.00, *p* < 0.001, *d* = −0.86). Moreover, current same-order trials yielded significantly smaller RTs when previous trials were also same-order trials compared to different-order trials (*M* = 67 ms; *t*(33) = −5.14, *p* < 0.001, *d* = −0.88). RTs in current different-order trials were also smaller following same-order trials compared to different-order trials (*M* = 30 ms; *t*(33) = −2.58, *p* = 0.014, *d* = −0.44). However, the modulation of this interaction by SESSION did not reach statistical significance, *F*(1, 16) = 4.21, *p* = 0.057, BF_01_ = 0.104, with Bayesian analysis providing only weak support for the null model. To sum up, regarding the separability hypothesis of dual-task coordination and coordination adjustment, the RT analysis reveals dual-task practice effects for task-order coordination, whereas evidence for practice effects in task-order coordination adjustment is less conclusive.

#### 3.1.2 Dual-task practice effects as reflected in errors

**Figure 4:**
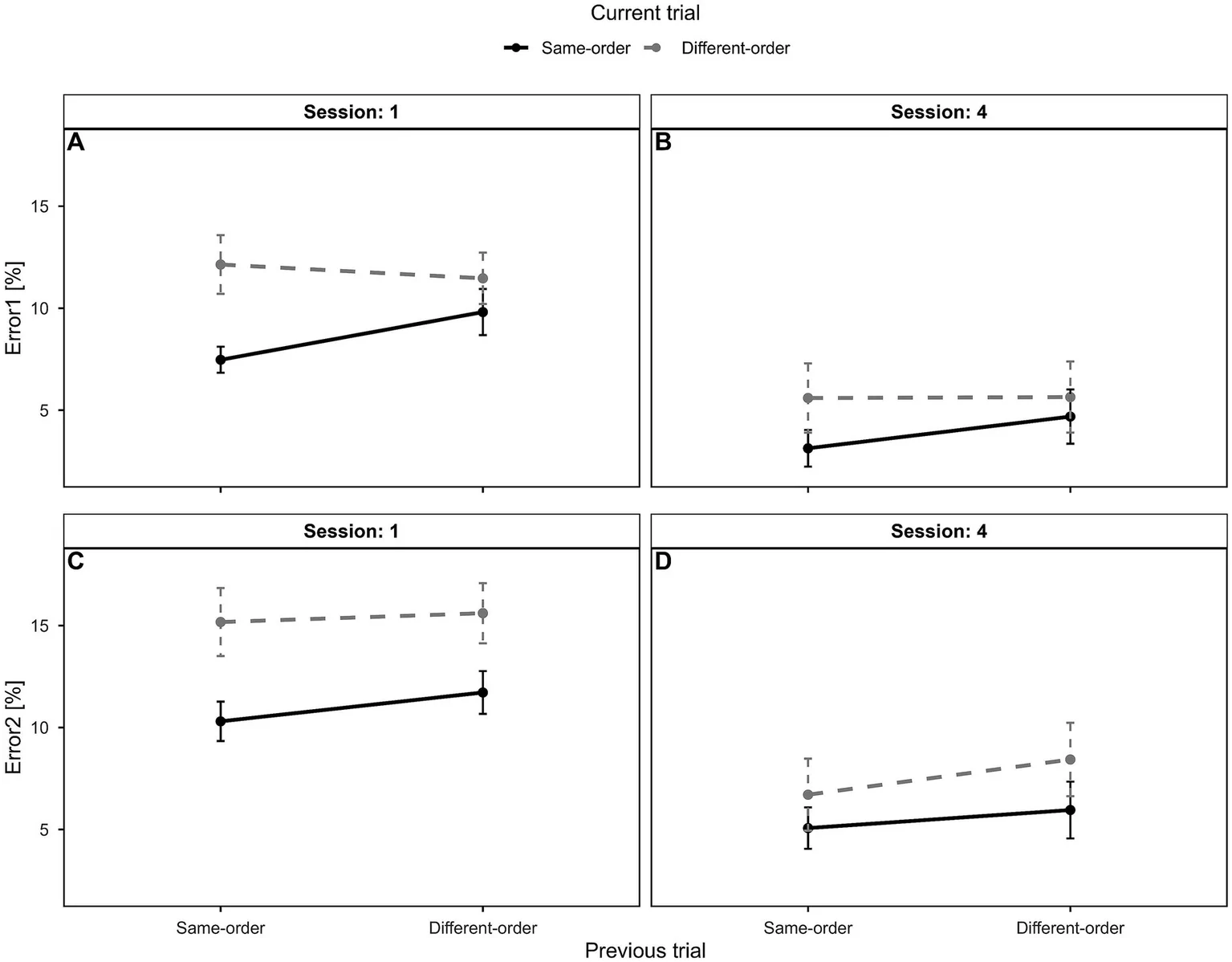
Error rates in the first (Error1) and second task (Error2) in percent for Session 1 **(A,C)** and Session 4 **(B,D)** in the dual-task practice group. Error rates are shown for same-order and different-order in the current trial and for same-order and different-order in the previous trial. Error bars represent standard errors of the mean.

The analysis of Error1 also revealed a significant main effect of CURRENT TRIAL, *F*(1, 16) = 12.33, *p* = 0.003, *ŋp2* = 0.435, BF_01_ = 0.063, indicating order-switch costs. As illustrated in Figures 4A,B, error rates were lower in same-order trials (*M* = 6.3%, SD = 3.6%) compared to different-order trials (*M* = 8.7%, SD = 5.7%). A significant main effect of PREVIOUS TRIAL also emerged, *F*(1, 16) = 7.12, *p* = 0.017, *ŋp2* = 0.308, BF_01_ = 0.633, reflecting generally lower error rates in the current trials when the previous ones were same-order trials (*M* = 7.1%, SD = 4.2%) relative to different-order trials (*M* = 7.9%, SD = 4.9%). Consistent with practice effects, a significant main effect of SESSION appeared, *F*(1, 16) = 28.06, *p* < 0.001, *ŋp2* = 0.637, BF_01_ = 0.003. Error rates were overall lower in Session 4 (*M* = 4.8%, SD = 5.7%) compared to Session 1 (*M* = 10.2%, SD = 4.2). Furthermore, CURRENT TRIAL was modulated by SESSION, *F*(1, 16) = 4.87, *p* = 0.042, *ŋp2* = 0.233, BF_01_ = 0.737. Order-switch costs decreased from Session 1 (*M* = 3.2%; *t*(16) = −3.68, *p* = 0.002, *d* = −0.89) to Session 4 (*M* = 1.7%; *t*(16) = −2.58, *p* = 0.020, *d* = −0.63), revealing improved task-order coordination during dual-task practice. There was no significant interaction between PREVIOUS TRIAL and SESSION, *F*(1, 16) < 0.01, *p* = 0.957, BF_01_ = 3.270, indicating that the reduction in error rates in current trials following same-order compared to different-order trials did not differ reliably between Session 1 and Session 4. Moreover, Error1 showed a significant interaction between CURRENT TRIAL and PREVIOUS TRIAL, *F*(1, 16) = 10.64, *p* = 0.005, *ŋp2* = 0.399, BF_01_ = 0.127. Order-switch costs in the current trials were significantly smaller following different-order trials (*M* = 1.3%; *t*(33) = −2.17, *p* = 0.038, *d* = −0.37) relative to following same-order trials (*M* = 3.6%; *t*(33) = −5.17, *p* < 0.001, *d* = −0.89). Additionally, error rates in current same-order trials were significantly smaller following same-order trials in comparison to different-order trials (*M* = 1.9%; *t*(33) = −3.94, *p* < 0.001, *d* = −0.68). In contrast, error rates in current different-order trials did not differ depending on whether the preceding trial was a different-order or a same-order trial (*t*(33) = 0.79, *p* = 0.433). However, this interaction was not significantly modulated by SESSION, *F*(1, 16) = 1.24, *p* = 0.283, BF_01_ = 1.391. Hence, there is no indication of a practice-related modulation of task-order coordination adjustment.

Analyzing Error2 similar to Error1, the main effect of CURRENT TRIAL was significant, *F*(1, 16) = 14.69, *p* = 0.001, *ŋp2* = 0.479, BF_01_ = 0.036, indicating the presence of order-switch costs. As depicted in Figures 4C,D, participants responded more accurate in same-order trials (*M* = 8.3%, SD = 3.8%) compared to different-order trials (*M* = 11.5%, SD = 5.9%). The effect of PREVIOUS TRIAL also emerged significance, *F*(1, 16) = 18.07, *p* < 0.001, *ŋp2* = 0.530, BF_01_ = 0.275, revealing overall lower error rates in the current trials when the previous ones were same-order trials (*M* = 9.3%, SD = 4.4%) relative to different-order trials (*M* = 10.4%, SD = 5.0%). SESSION also exerted influence, *F*(1, 16) = 29.45, *p* < 0.001, *ŋp2* = 0.648, BF_01_ = 0.003, reflecting general practice effects. Participants made fewer errors in Session 4 (*M* = 6.5%, SD = 5.8%) compared to Session 1 (*M* = 13.2%, SD = 4.8%). Moreover, CURRENT TRIAL was modulated by SESSION, *F*(1, 16) = 7.73, *p* = 0.013, *ŋp2* = 0.326, BF_01_ = 0.299. In line with the assumption of improved task-order coordination during practice, order-switch costs decreased from Session 1 (*M* = 4.4%; *t*(16) = −4.29, *p* < 0.001, *d* = −1.04) to Session 4 (*M* = 2.1%; *t*(16) = −2.43, *p* = 0.027, *d* = −0.59). As in Error1, there was no significant interaction between PREVIOUS TRIAL and SESSION for Error2, *F*(1, 16) = 0.23, *p* = 0.636, BF_01_ = 2.838. Additionally, there was no significant interaction between CURRENT TRIAL and PREVIOUS TRIAL, *F*(1, 16) = 0.01, *p* = 0.940, BF_01_ = 2.897, indicating that order-switch costs in the current trial did not reliably differ depending on whether the preceding trial involved a same-order or different-order trial. Moreover, no significant three-way interaction with SESSION was observed, *F*(1, 16) = 1.21, *p* = 0.288, BF_01_ = 1.414. Thus, there is no indication of a practice-related modulation of task-order coordination adjustment. In sum, with regard to the separability hypothesis of dual-task coordination and coordination adjustment, the error analysis reveals dual-task practice effects for task-order coordination, but no corresponding practice effect for task-order coordination adjustment.

#### 3.1.3 Dual-task practice effects as reflected in response reversals

**Figure 5:**
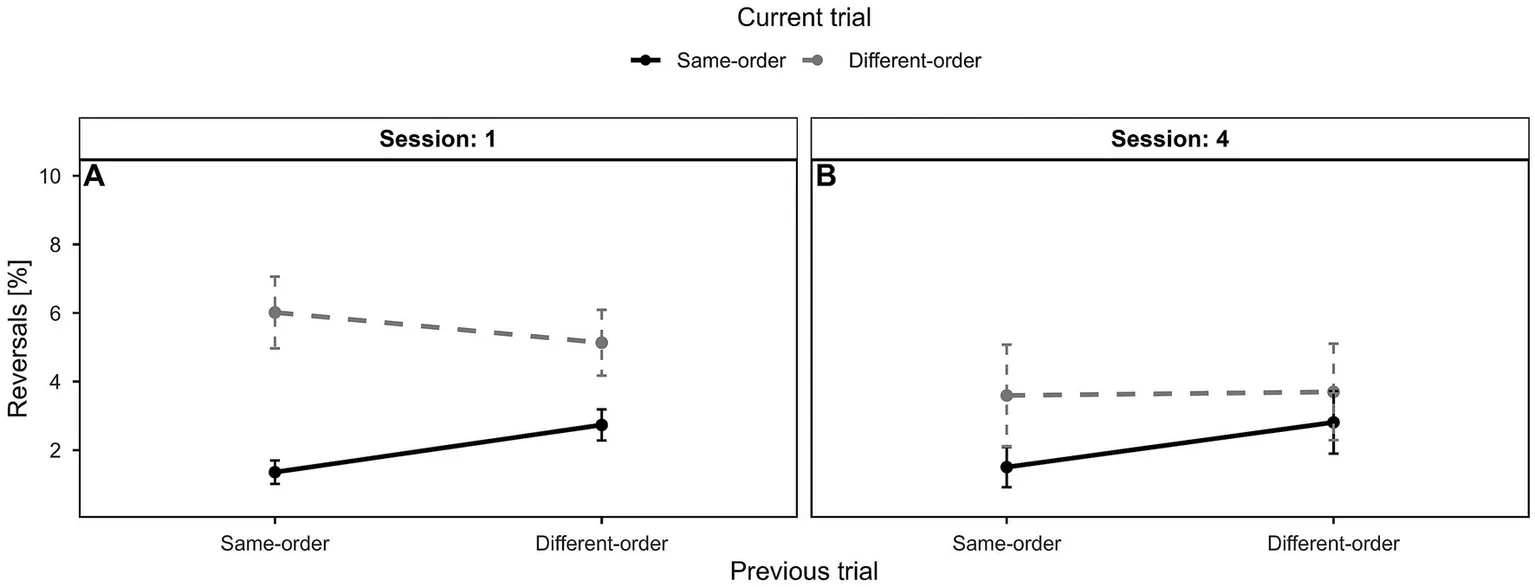
Response reversal rates in percent for Session 1 **(A)** and Session 4 **(B)** in the dual-task practice group. Reversal rates are shown for same-order and different-order in the current trial and for same-order and different-order in the previous trial. Error bars represent standard errors of the mean.

The analysis of response reversals revealed a significant main effect of CURRENT TRIAL, *F*(1, 16) = 14.70, *p* = 0.001, *ŋp2* = 0.479, BF_01_ = 0.037. As shown in Figures 5A,B, response reversals in current trials were lower in same-order trials (*M* = 2.1%, SD = 2.1%) compared to different-order trials (*M* = 4.6%, SD = 4.6%). A significant main effect of PREVIOUS TRIAL also emerged, *F*(1, 16) = 5.10, *p* = 0.038, *ŋp2* = 0.242, BF_01_ = 1.495, with Bayesian analysis providing anecdotal evidence in favor of the null model. Descriptively, there were overall lower response reversal rates in the current trials when the previous ones were same-order trials (*M* = 3.1%, SD = 3.3%) relative to different-order trials (*M* = 3.6%, SD = 3.4%). Given the low response reversal rates, floor effects may have constrained variability and should be considered when interpreting the results. There was no significant main effect of SESSION, *F*(1, 16) = 1.73, *p* = 0.207, BF_01_ = 1.280, indicating no general practice effect in response reversals. However, CURRENT TRIAL was modulated by SESSION, *F*(1, 16) = 12.52, *p* = 0.003, *ŋp2* = 0.439, BF_01_ = 0.085, reflecting a decrease in order-switch costs from Session 1 (*M* = 3.5%; *t*(16) = −4.95, *p* < 0.001, *d* = −1.20) to Session 4 (*t*(16) = −2.08, *p* = 0.054). This interaction was consistent with our assumption of a practice-related improvement of task-order coordination. There was no significant interaction between PREVIOUS TRIAL and SESSION, *F*(1, 16) = 0.60, *p* = 0.451, BF_01_ = 2.529, indicating that the reduction in response reversal rates in current trials following same-order compared to different-order trials did not differ between Session 1 and Session 4. Finally, our analysis revealed a significant interaction between CURRENT TRIAL and PREVIOUS TRIAL, *F*(1, 16) = 13.20, *p* = 0.002, *ŋp2* = 0.452, BF_01_ = 0.124. Order-switch costs in the current trials were significantly smaller following different-order trials (*M* = 1.6%; *t*(33) = −3.31, *p* = 0.002, *d* = −0.57) relative to following same-order trials (*M* = 3.4%; *t*(33) = −5.03, *p* < 0.001, *d* = −0.89). Further, response reversal rates in current same-order trials were significantly smaller following same-order trials relative to different-order trials (*M* = 1.3%; *t*(33) = −4.71, *p* < 0.001, *d* = −0.81). In contrast, response reversal rates in current different-order trials did not differ depending on whether the preceding trial was a different-order or a same-order trial (*t*(33) = 0.90, *p* = 0.376). However, this interaction did not significantly depend on SESSION, *F*(1, 16) = 0.86, *p* = 0.367, BF_01_ = 1.733. Thus, there is no indication of a practice-related modulation of task-order coordination adjustment. In sum, concerning the separability hypothesis of dual-task coordination and coordination adjustment, the response reversal analysis indicates dual-task practice effects on task-order coordination, whereas task-order coordination adjustment appears largely unaffected by practice.

### 3.2 Between-subject comparison of Session 4 in the dual-task and single-task practice groups

Remarkably, the baseline comparison of the initial dual-task block in Session 1 revealed a significant main effect of GROUP regarding RT1, *F*(1, 40) = 4.15, *p* = 0.048, BF_01_ = 0.854. The single-task practice group showed lower RTs at baseline (*M* = 1,156 ms, SD = 354 ms) compared to the dual-task practice group (*M* = 1,365 ms, SD = 311 ms). In contrast, the analysis of RT2 showed no significant main effect of GROUP, *F*(1, 40) = 1.80, *p* = 0.187, BF_01_ = 1.535. Descriptively, the single-task practice group showed lower RTs at baseline (*M* = 1,458 ms, SD = 394 ms) compared to the dual-task practice group (*M* = 1,611 ms, SD = 349 ms). Importantly, RT1 and RT2 showed no interaction of GROUP and CURRENT TRIAL, *Fs*(1, 40) ≤ 0.68, *ps* ≥ 0.415, BFs_01_ ≥ 2.689, which would have indicated differences in task-order coordination prior practice. Similarly, analyses of Error1 and Error2 resulted in no main effect of GROUP, *Fs*(1, 40) ≤ 0.48, *ps* ≥ 0.492, BFs_01_ ≥ 1.804, or interaction with CURRENT TRIAL, *Fs*(1, 40) ≤ 2.85, *ps* ≥ 0.099, BFs_01_ ≥ 1.800. Response reversals also showed no main effect of GROUP, *F*(1, 40) = 0.59, *p* = 0.445, BF_01_ = 1.470, or interaction with CURRENT TRIAL, *F*(1, 40) = 0.68, *p* = 0.414, BF_01_ = 4.277. Hence, differences in task-order coordination after dual-task and single-task practice cannot be explained with differences in task-order coordination at the beginning of practice.

#### 3.2.1 Dual-task versus single-task practice effects as reflected in RTs

**Figure 6:**
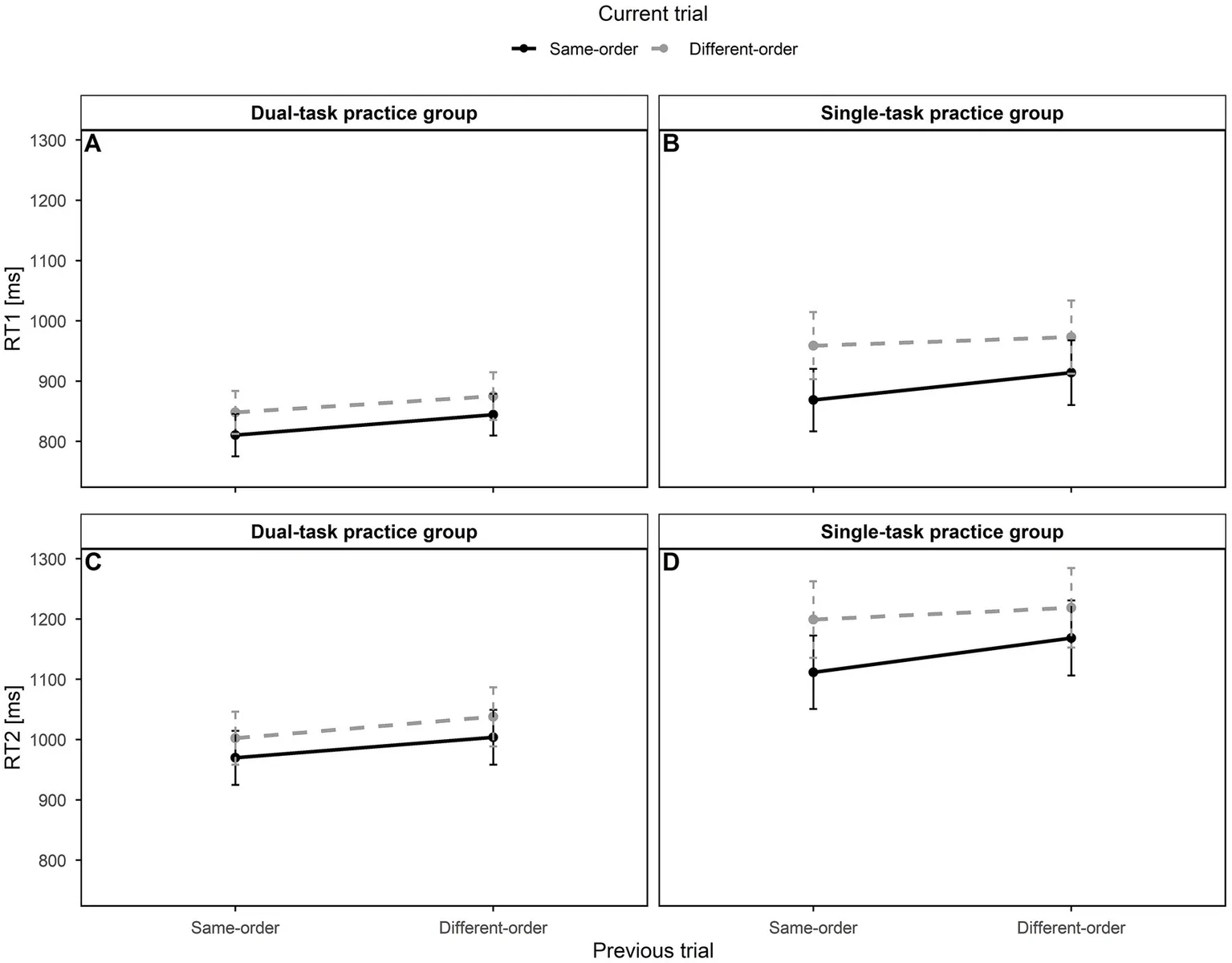
Reaction times in the first (RT1) and second task (RT2) in ms for the dual-task practice group **(A,C)** and the single-task practice group **(B,D)** in Session 4. RTs are shown for same-order and different-order in the current trial and for same-order and different-order in the previous trial. Error bars represent standard errors of the mean.

Analyzing RT1, a significant main effect of CURRENT TRIAL emerged, *F*(1, 40) = 56.73, *p* < 0.001, *ŋp2* = 0.586, BF_01_ < 0.001, indicating order-switch costs. As shown in Figures 6A,B, participants responded faster in same-order trials (*M* = 858 ms, SD = 200 ms) compared to different-order trials (*M* = 911 ms, SD = 222 ms). The analysis of RT1 also revealed a significant main effect of PREVIOUS TRIAL, *F*(1, 40) = 40.17, *p* < 0.001, *ŋp2* = 0.501, BF_01_ < 0.001, reflecting generally shorter RTs in the current trials when the previous ones were same-order trials (*M* = 870 ms, SD = 205 ms) compared to different-order trials (*M* = 900 ms, SD = 216 ms). There was no significant effect for GROUP, *F*(1, 40) = 1.71, *p* = 0.198, BF_01_ = 2.045, indicating no reliable difference in overall RTs for Task 1. However, CURRENT TRIAL was modulated by GROUP, *F*(1, 40) = 7.79, *p* = 0.008, *ŋp2* = 0.163, BF_01_ = 0.249. Order-switch costs were smaller in the dual-task practice group (*M* = 34 ms; *t*(21) = −3.81, *p* = 0.001, *d* = −0.81) than in the single-task practice group (*M* = 75 ms; *t*(19) = −6.49, *p* < 0.001, *d* = −1.45), indicating improved task-order coordination following dual-task practice compared with the effects of single-task practice. A modulation of PREVIOUS TRIAL by GROUP remained non-significant, *F*(1, 40) = 0.01, *p* = 0.940, BF_01_ = 4.293, indicating that the reduction in RT in current trials following same-order compared to different-order trials did not differ between the two groups. Remarkably, there was no significant interaction between CURRENT TRIAL and PREVIOUS TRIAL, *F*(1, 40) = 2.17, *p* = 0.149, BF_01_ = 1.313, demonstrating that order-switch costs in the current trial did not differ depending on whether the preceding trial involved a same-order or different-order trial. Further, no significant three-way interaction with GROUP was observed, *F*(1, 40) = 1.71, *p* = 0.198, BF_01_ = 1.410. Thus, there is no evidence demonstrating that the different practice conditions modulated task-order coordination adjustment differently.

Analyzing RT2 similar to RT1, the main effect of CURRENT TRIAL was again significant, *F*(1, 40) = 48.96, *p* < 0.001, *ŋp2* = 0.550, BF_01_ < 0.001, indicating order-switch costs. As visualized in Figures 6C,D, RTs were lower in same-order trials (*M* = 1,060 ms, SD = 252 ms) compared to different-order trials (*M* = 1,110 ms, SD = 267 ms). The effect of PREVIOUS TRIAL was also significant, *F*(1, 40) = 49.82, *p* < 0.001, *ŋp2* = 0.555, BF_01_ < 0.001, reflecting overall faster responses in the current trials when the previous ones were same-order trials (*M* = 1,067 ms, SD = 254 ms) relative to different-order trials (*M* = 1,103 ms, SD = 264 ms). RTs for Task 2 also differed for GROUP, *F*(1, 40) = 5.04, *p* = 0.030, *ŋp2* = 0.112, BF_01_ = 1.348, with Bayesian analysis providing anecdotal evidence in favor of the null model. Descriptively, participants of the dual-task practice group (*M* = 1,004 ms, SD = 213 ms) reacted faster than participants of the single-task practice group (*M* = 1,175 ms, SD = 279 ms). Moreover, CURRENT TRIAL was modulated by GROUP, *F*(1, 40) = 5.92, *p* = 0.020, *ŋp2* = 0.129, BF_01_ = 0.581, indicating lower order-switch costs in the dual-task (*M* = 33 ms; *t*(21) = −3.87, *p* < 0.001, *d* = −0.83) compared to the single-task practice group (*M* = 69 ms; *t*(19) = −5.71, *p* < 0.001, *d* = −1.28). This result is consistent with the assumption of improved task-order coordination following dual-task practice compared with the effects of single-task practice. The interaction between PREVIOUS TRIAL and GROUP remained non-significant, *F*(1, 40) = 0.11, *p* = 0.746, BF_01_ = 4.135. As for RT1, neither a significant interaction between CURRENT TRIAL and PREVIOUS TRIAL, *F*(1, 40) = 1.54, *p* = 0.222, BF_01_ = 4.216, nor a three-way interaction with GROUP, *F*(1, 40) = 1.54, *p* = 0.194, BF_01_ = 0.810, was observed, although Bayesian analysis provided only weak support for the null hypothesis in the latter interaction. Overall, the RT analysis indicates that dual-task practice, relative to single-task practice, improves task-order coordination, while providing less clear evidence for changes in task-order coordination adjustment.

#### 3.2.2 Dual-task versus single-task practice effects as reflected in errors

**Figure 7:**
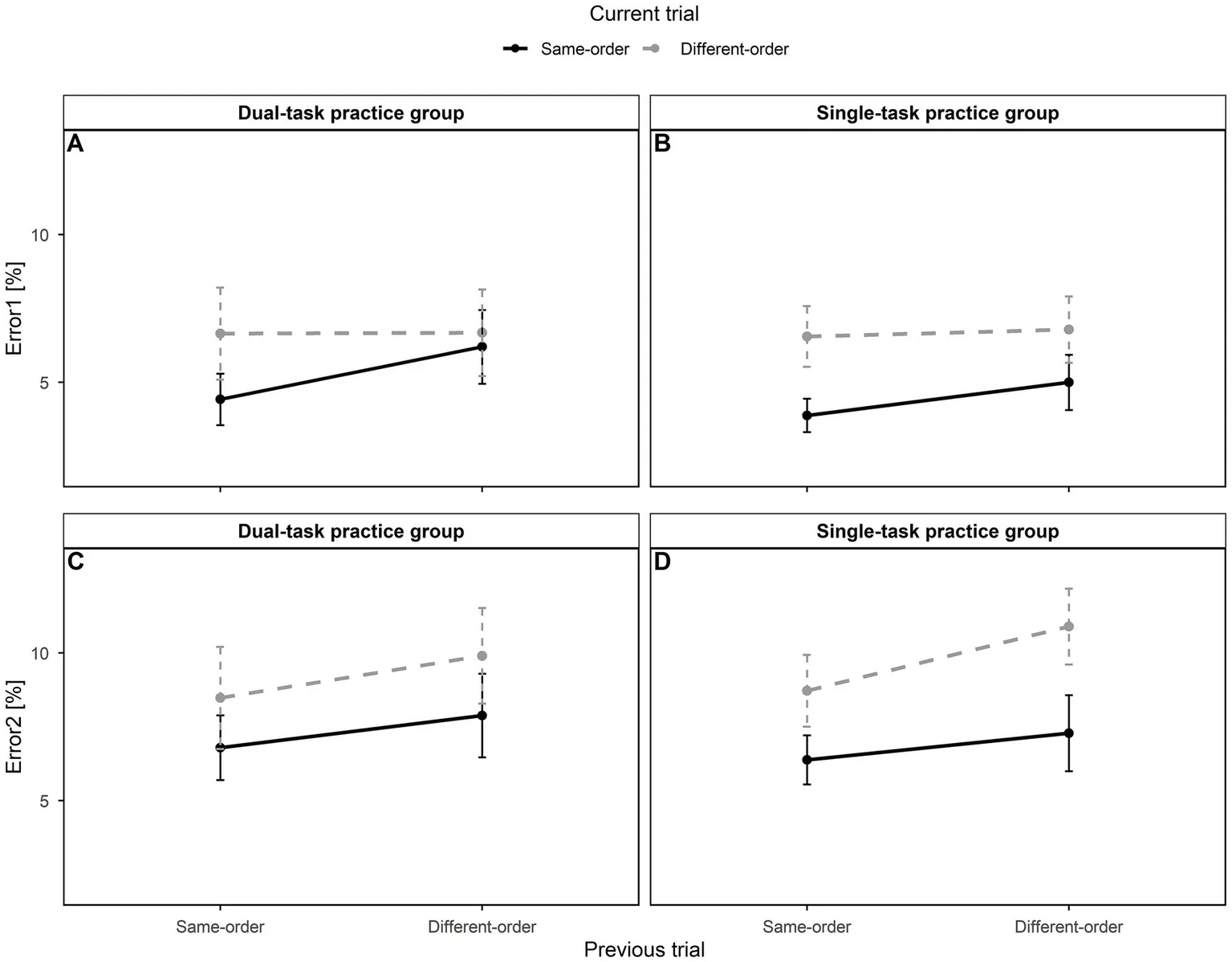
Error rates in the first (Error1) and second task (Error2) in percent for the dual-task practice group **(A,C)** and the single-task practice group **(B,D)** in Session 4. Error rates are shown for same-order and different-order in the current trial and for same-order and different-order in the previous trial. Error bars represent standard errors of the mean.

The Error1 analysis yielded a significant main effect of CURRENT TRIAL, *F*(1, 40) = 15.56, *p* < 0.001, *ŋp2* = 0.280, BF_01_ = 0.013, indicating order-switch costs. As shown in Figures 7A,B, error rates were lower in same-order trials (*M* = 4.9%, SD = 4.1%) compared to different-order trials (*M* = 6.7%, SD = 5.8%). The analysis of Error1 also demonstrated a significant main effect of PREVIOUS TRIAL, *F*(1, 40) = 6.45, *p* = 0.015, *ŋp2* = 0.139, BF_01_ = 0.827, reflecting lower error rates in the current trials when the previous ones were same-order trials (*M* = 5.4%, SD = 4.5%) compared to different-order trials (*M* = 6.2%, SD = 5.3%). As with RT1, the analysis of Error1 revealed no significant effect of GROUP, *F*(1, 40) = 0.08, *p* = 0.774, BF_01_ = 2.365, indicating that overall error rates for Task 1 did not reliably differ between the two groups. Additionally, the interaction between CURRENT TRIAL and GROUP was not significant, *F*(1, 40) = 0.93, *p* = 0.340, BF_01_ = 1.270, indicating no reliable differences in order-switch costs between groups and thus no different impact of the two practice conditions on task-order coordination. There was also no significant interaction between PREVIOUS TRIAL and GROUP, *F*(1, 40) = 0.13, *p* = 0.716, BF_01_ = 1.343. Moreover, the interaction between CURRENT TRIAL and PREVIOUS TRIAL did not reach statistical significance, *F*(1, 40) = 3.12, *p* = 0.085, BF_01_ = 0.674, although Bayesian analysis indicated only weak evidence in favor of the null hypothesis. Similarly, the three-way interaction with GROUP was non-significant, *F*(1, 40) = 0.33, *p* = 0.566, BF_01_ = 2.030. Thus, there is no evidence in the Error1 data showing that the different practice conditions modulated task-order coordination adjustment differently.

Analysis of Error2 revealed a comparable pattern of results. The main effect of CURRENT TRIAL was significant, *F*(1, 40) = 26.40, *p* < 0.001, *ŋp2* = 0.398, BF_01_ < 0.001, again reflecting the presence of order-switch costs. As depicted in Figures 7C,D, error rates were lower in same-order trials (*M* = 7.1%, SD = 5.2%) compared to different-order trials (*M* = 9.5%, SD = 6.6%). The effect of PREVIOUS TRIAL also emerged significance, *F*(1, 40) = 16.91, *p* < 0.001, *ŋp2* = 0.297, BF_01_ = 0.030, indicating lower error rates in the current trials when the previous ones were same-order trials (*M* = 7.6%, SD = 5.4%) relative to different-order trials (*M* = 9.0%, SD = 6.2%). No significant effects were observed for GROUP, *F*(1, 40) < 0.01, *p* = 0.976, BF_01_ = 1.934, nor for its interactions with CURRENT TRIAL, *F*(1, 40) = 1.42, *p* = 0.240, BF_01_ = 1.520, or PREVIOUS TRIAL, *F*(1, 40) = 0.17, *p* = 0.681, BF_01_ = 1.171. As with Error1, the interaction between CURRENT TRIAL and PREVIOUS TRIAL was also not significant for Error2, *F*(1, 40) = 1.21, *p* = 0.278, BF_01_ = 2.444. Similarly, the three-way interaction including GROUP did not reach significance, *F*(1, 40) = 0.42, *p* = 0.522, BF_01_ = 1.593. Thus, the Error2 data revealed no evidence for the assumption that the different practice conditions affected task-order coordination and task-order coordination adjustment differently. To sum up, the error analyses provide no evidence that task-order coordination or task-order coordination adjustment improved more following dual-task than single-task practice.

#### 3.2.3 Dual-task versus single-task practice effects as reflected in response reversals

**Figure 8:**
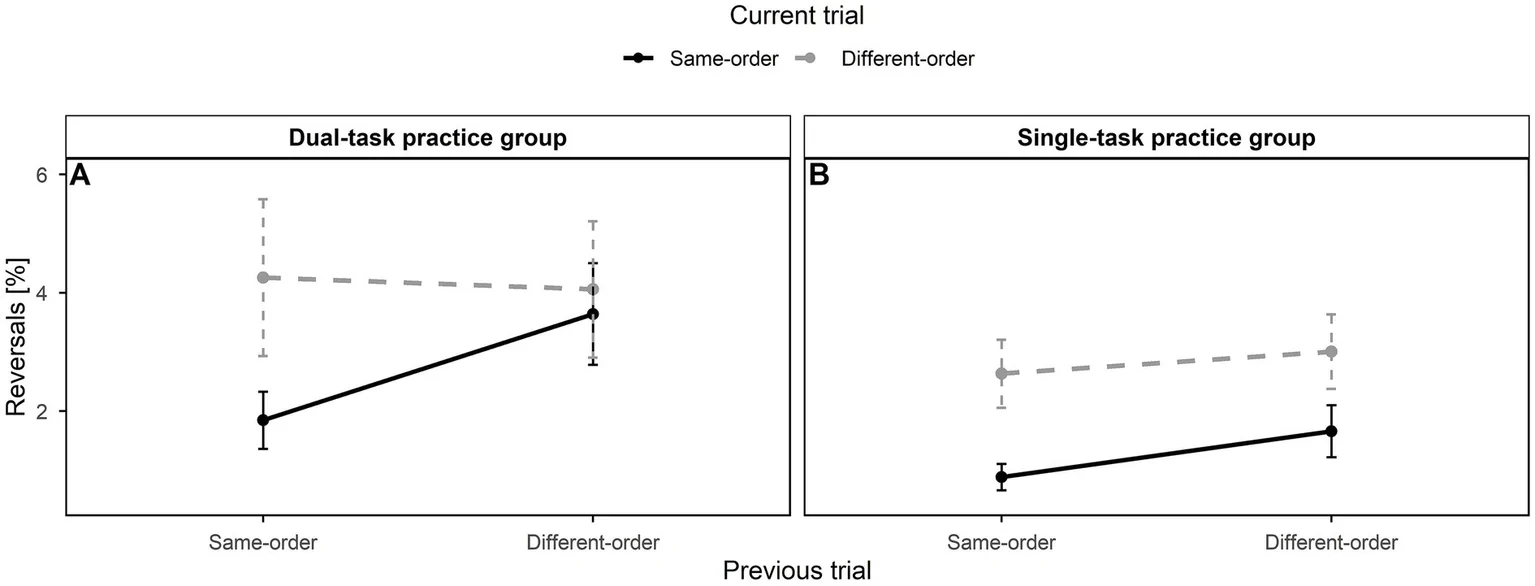
Response reversal rates in percent for the dual-task practice group **(A)** and the single-task practice group **(B)** in Session 4. Reversal rates are shown for same-order and different-order in the current trial and for same-order and different-order in the previous trial. Error bars represent standard errors of the mean.

The analysis of response reversals showed a significant main effect of CURRENT TRIAL, *F*(1, 40) = 13.87, *p* < 0.001, *ŋp2* = 0.257, BF_01_ = 0.017. As visualized in Figures 8A,B, response reversals were lower in same-order trials (*M* = 2.0%, SD = 2.5%) compared to different-order trials (*M* = 3.5%, SD = 4.4%). The main effect of PREVIOUS TRIAL was also significant, *F*(1, 40) = 9.44, *p* = 0.004, *ŋp2* = 0.191, BF_01_ = 0.519, indicating that response reversal rates in the current trials were generally lower when the preceding trial was a same-order trial (*M* = 2.4%, SD = 3.2%) compared to a different-order trial (*M* = 3.1%, SD = 3.6%). Again, given the low response reversal rates, floor effects may have constrained variability and should be considered when interpreting the results. GROUP showed no significant effect, *F*(1, 40) = 1.89, *p* = 0.177, BF_01_ = 1.257, and neither did its interactions with CURRENT TRIAL, *F*(1, 40) = 0.03, *p* = 0.868, BF_01_ = 3.251, or with PREVIOUS TRIAL, *F*(1, 40) = 0.25, *p* = 0.618, BF_01_ = 3.903. The interaction between CURRENT TRIAL and PREVIOUS TRIAL was significant, *F*(1, 40) = 4.22, *p* = 0.047, *ŋp2* = 0.095, BF_01_ = 0.324, showing smaller order-switch costs in current trials following different-order trials (*M* = 0.9%; *t*(41) = −2.10, *p* = 0.042, *d* = −0.32) than following same-order trials (*M* = 2.1%; *t*(41) = −3.74, *p* < 0.001, *d* = −0.58). Additionally, response reversal rates in current same-order trials were significantly smaller following same-order trials relative to different-order trials (*M* = 1.3%; *t*(41) = −3.92, *p* < 0.001, *d* = −0.61). In contrast, there was no significant response reversal rate difference in current different-order trials when the previous trials were same-order and different-order trials, *t*(41) = −0.18, *p* = 0.856. However, the three-way interaction with GROUP remained non-significant, *F*(1, 40) = 1.86, *p* = 0.180, BF_01_ = 1.069. Thus, the response reversal data revealed no evidence for the assumption that the different practice conditions affected task-order coordination and task-order coordination adjustment differently.

## 4 Discussion

The aim of the present study was to examine the separability between dual-task coordination and coordination adjustment in the context of cued task-order coordination following practice. The results indicate solid practice-related improvements in task-order coordination. However, evidence for practice effects in task-order coordination adjustment remains very limited (see Table 3). Thus, the differential pattern of practice-related effects offers limited support for the separability of these processes.

### 4.1 Cued task-order coordination

Across Session 1 (dual-task practice group) and Session 4 (both groups), performance in different-order trials was generally impaired relative to performance in same-order trials, resulting in order-switch costs and indicating task-order coordination (Kübler et al., 2018; Strobach, 2024a; Strobach et al., 2021b; Szameitat et al., 2006). This pattern can be explained by the assumption that individuals actively maintain task-order sets in working memory (Hirsch and Koch, 2024; Kübler et al., 2022a). In same-order trials, the task-order set from the previous trial can be reused, whereas different-order trials require the implementation of a new task-order set. This process, along with the potential inhibition of the previously active task-order set, is time-consuming and results in order-switch costs. Furthermore, in contrast to same-order trials, different-order trials are not only characterized by a switch in task order but also by a switch of the corresponding task-order cue. Consequently, observed performance differences (i.e., order-switch costs) may also reflect differences in cue processing between cue-switch and cue-repetition trials, rather than reflecting the processing of task-order sets themselves (Mayr and Kliegl, 2003; Steinhauser et al., 2021). However, related research on task-pair coordination in dual tasks reported (task-pair) switch costs that even occurred when cue switching is controlled for, suggesting that task-set switching per se entails performance costs that cannot be reduced to costs of cue switching (Hirsch et al., 2021). In addition, most studies on task-order coordination did not even implement order cues in their experiment designs (Kübler et al., 2018; Strobach, 2024a; Strobach et al., 2021b; Szameitat et al., 2006) and nevertheless still observed order-switch costs. Therefore, order-switch costs cannot be attributed solely to a switch in the cue.

Moreover, these order-switch costs were reduced in Session 4 compared to Session 1, indicating practice effects on cued task-order coordination. Dual-task practice may have encouraged participants to adopt a coordination strategy that facilitates the development of dual-task coordination skills, such as efficient cognitive resource allocation and task scheduling (Strobach, 2020, 2024b; Strobach and Schubert, 2017a). These skills might lead to a more efficient and conjoint instantiation of relevant task information (i.e., the appropriate task-order set) in working memory during dual-task trials (Schubert et al., 2024; Strobach et al., 2014). In particular, they may facilitate the switching process involved in inhibiting a previously active task-order set and activating a new task-order set in working memory (Hirsch and Koch, 2024; Kübler et al., 2022a; Lien et al., 2003). As these processes are primarily required in different-order trials, their facilitation reduces performance differences between different-order and same-order trials, resulting in smaller order-switch costs following practice. Moreover, the association between the cue and the task-order set may strengthen with practice, thereby fastening the loading of the adequate cue information in working memory. The impact of the later type of information is elegantly demonstrated by De Jong (1995), who manipulated task-order cues to be either consistent or inconsistent with the subsequent stimulus presentation order. The RTs significantly increased in the inconsistent relative to the consistent condition, suggesting that the instantiation of task-relevant information through cues drastically affects performance in dual-task contexts with varying task orders. Thus, practice may strengthen the association from the cue to the task-order set, thereby reducing order-switch costs (Mayr and Kliegl, 2003).

However, these findings are limited by the absence of evidence for superior task-order coordination following dual-task practice compared to single-task practice in terms of error rates and response reversal rates as indicated by the between-subject comparisons (see Sections 3.2.2 and 3.2.3). Therefore, improvements in task-order coordination may alternatively be explained by general experience with the individual component tasks. This experience may lead to practice-related shortening of task-processing stages (see Figure 1) and, consequently, to decreased interference (Ruthruff et al., 2006; Schubert et al., 2026; Van Selst et al., 1999), or even to the complete automatization of component-task processing, thereby eliminating processes that compete for limited capacities such as the central bottleneck (Maquestiaux et al., 2008; Ruthruff et al., 2006). However, these accounts would also predict comparable practice effects on RTs following both dual-task and single-task practice, which is not supported by the data. Specifically, order-switch costs in RTs were smaller following dual-task practice than single-task practice, which indicates better task-order coordination in terms of RTs. Thus, extending Darnstaedt et al. (2024), the present findings reveal that varying task-order dual-task practice improves task-order coordination in RTs not only relative to fixed task-order practice, but also compared with single-task practice. Moreover, this effect is not limited to dual-task settings in which task order is exclusively determined by the sequence of stimulus presentation (Strobach, 2024b), but also emerges in paradigms with explicit task-order cues.

### 4.2 Cued task-order coordination adjustment

With the exception of Error2, the within-subject comparisons (see Section 3.1) generally revealed reduced order-switch costs following a different-order Trial N-1, compared to a same-order Trial N-1, indicating switch adjustment effects (Strobach et al., 2021a, 2023; Strobach and Wendt, 2022). These findings of adjustments in cued task-order coordination, previously reported by Steinhauser et al. (2024), are thus basically replicated. Such effects can be explained by sequential adjustments of cognitive control. According to the conflict monitoring theory proposed by Botvinick et al. (2001, 2004), the cognitive system continuously monitors ongoing task and response processing to detect conflicts. When a conflict is identified, cognitive control is increased, leading to reduced processing of irrelevant information and/or enhanced processing of relevant information. Accordingly, encountering a different-order Trial N-1, as opposed to a same-order Trial N-1, increases cognitive control, which in turn facilitates the inhibition of the previous task-order set and/or the activation of a new task-order set, thereby reducing order-switch costs in Trial N (Hirsch and Koch, 2024; Kübler et al., 2022a; Strobach et al., 2021a). Moreover, different-order Trials N-1, compared to same-order Trials N-1, are also characterized by a switch in the order cue, which may itself be experienced as a form of conflict, thereby increasing cognitive control and potentially facilitating cue processing in Trial N. To our knowledge, a study investigating cued task-order coordination adjustment while controlling for cue switching [similar to the design used by Hirsch et al. (2021) for task-pair coordination], in order to clarify the influence of cue switching on sequential adjustment, is still lacking. However, most studies on sequential adjustments did not include explicit order cues in their experimental designs (Strobach et al., 2021a, 2023; Strobach and Wendt, 2022), yet still observed switch adjustment effects. Thus, similar to order-switch costs, switch adjustment effects may not be explained solely in terms of cue switching.

Although practice effects on cued task-order coordination led to generally reduced order-switch costs (see Section 4.1), there is no evidence that dual-task practice affects the switch adjustment effect in error rates or response reversal rates, and only limited evidence in RTs. Overall, the evidence for dual-task practice on cued task-order coordination adjustment remains weak. Therefore, the absence of a clear modulation in switch adjustment effects across practice sessions indicates that a strategy of dynamic cognitive control adjustment may be maintained even with extensive practice. This sustained adjustment may be driven by constantly high levels of conflict induced by different-order Trials N-1, which in turn reduces order-switch costs in Trial N (Botvinick et al., 2001, 2004; Strobach et al., 2021a), even after practice, similar to findings reported in task-switching paradigms (Strobach et al., 2020). Following practice, a stronger association between the cue and the corresponding task-order set in a different-order Trial N-1 may facilitate the loading of appropriate information into working memory (Mayr and Kliegl, 2003). However, the switch in task order (or cues) is still detected as a conflict, resulting in switch adjustment effects.

Overall, these findings are in line with Strobach (2024b) and provide limited evidence for the separability hypothesis proposed in the framework of Figure 1, according to which dual-task coordination (e.g., task-order coordination) and dual-task coordination adjustment show differentiable result patterns following practice. While the present results demonstrate clear practice effects on task-order coordination, evidence for practice effects on task-order coordination adjustment is less conclusive.

### 4.3 Limitations and outlook

In contrast to previous studies, reporting a more clear dissociation between dual-task coordination and coordination adjustment (Strobach, 2024a; Strobach et al., 2021a, 2023; Strobach and Wendt, 2022), the present findings suggest that this separability is less robust under the current experimental practice condition. To clarify this issue, future studies investigating task-order coordination and its adjustment are required. One promising non-practice approach is to further examine the role of working memory as a potential mechanism underlying dual-task coordination. Strobach et al. (2021a) found no evidence that increasing working memory load via an increase in task-set size affected either task-order coordination or task-order coordination adjustment. However, Kübler et al. (2022b) demonstrated that increasing working memory load via an additional updating task did influence task-order coordination and reduced order-switch costs, indicating that task-order sets and thus task-order coordination depend on working memory resources. Thus, manipulating working memory through updating tasks may represent a promising avenue for future research on the separability of dual-task coordination and coordination adjustment.

Moreover, the present study focused on mechanisms underlying improved task-order coordination mainly in the context of a central bottleneck model. In this context, it is important to consider alternative accounts of capacity limitation models that allow for parallel central processing (i.e., capacity sharing models, Tombu and Jolicœur, 2003; Wu and Wang, 2024). Given the very short response-selection stages of Task 1 and Task 2 after practice, capacity sharing models and strict bottleneck models would make largely similar predictions regarding the effects of the present single-task and dual-task practice procedure. In capacity sharing accounts, the shortening of the response-selection stages of both tasks following practice (Ruthruff et al., 2006; Schubert et al., 2026; Van Selst et al., 1999) would reduce the duration of capacity sharing between tasks. Likewise, in bottleneck models, shorter response-selection stages would reduce the time spent waiting at the bottleneck. Consequently, both accounts predict substantially reduced dual-task interference following extensive practice. To distinguish between the capacity sharing and central bottleneck accounts, it is informative to examine the effects of varying the SOA. Capacity sharing models predict that reducing the SOA should impair RT1, because the response-selection stages of both tasks increasingly overlap and must share limited central processing resources. In contrast, the central bottleneck model predicts no effect of SOA on RT1, as the response-selection stage of Task 2 cannot begin until response selection for Task 1 has been completed. The present study provides no such SOA manipulation to test the differential RT1 predictions of capacity sharing models and bottleneck models. However, within the context of investigating task-order coordination and coordination adjustment, Strobach et al. (2021a) compared dual-task performance using SOAs of 400 ms and 800 ms. They found no effect of SOA on RT1, consistent with the central bottleneck account. Thus, the RT data of an associated study resulted in evidence that is rather consistent with the bottleneck model and its underlying theoretical mechanisms.

Alternatively, practice effects may be explained by the integration of two tasks into a single “super task” (Hazeltine et al., 2002). According to this account, two separate response-selection processes are assumed at Session 1, whereas practice leads to a compound response-selection process for both tasks in Session 4. However, this account would predict the absence of order-switch costs and, consequently, no switch adjustment effects following practice, as the temporal order of response-selection stages would no longer be relevant within an integrated response-selection stage of a super task. In contrast to these predictions, the present results show order-switch costs and switch adjustment effects in Session 4 (see Section 3.1). Moreover, previous evidence suggests that response–response conflicts persists even in practiced setting (Böer et al., 2026) and that motor-motor dual tasks of the type used in the present study are not integrated through practice (Hazeltine et al., 2002; Strobach and Schubert, 2017b). Taken together, there is thus no empirical support for task integration in the present data. However, future studies should further elaborate on the specific characteristics of the processes that are order controlled and where this control is adjusted.

### 4.4 Summary

The present study demonstrated both cued task-order coordination and task-order coordination adjustment within a dual-task context. Overall, the findings indicate practice-related improvements in task-order coordination, whereas evidence of practice effects on coordination adjustment remains limited. Therefore, our study provides some evidence that supports the separability hypothesis. Thus, to further clarify the separability of task-order coordination and its adjustment processes, additional research is required.

## Data Availability

The datasets presented in this study can be found in online repositories. The names of the repository/repositories and accession number(s) can be found in the article/supplementary material.

## Footnotes

1 The established study of Strobach (2024b) did not reveal a significant interaction between CURRENT TRIAL, PREVIOUS TRIAL, and GROUP. Consequently, this interaction could not be used to derive an effect size for the *a priori* power analysis in the present study. To investigate differential practice effects on task-order coordination following dual-task versus single-task practice, the effect size was based on the corresponding interaction of CURRENT TRIAL and GROUP reported by Strobach (2024b).
